# Association Between Pulse Pressure With All-Cause and Cardiac Mortality in Acute Coronary Syndrome: An Observational Cohort Study

**DOI:** 10.3389/fcvm.2022.930755

**Published:** 2022-07-13

**Authors:** Man Wang, Wen Su, Chun-Yan Jiang, Wei-Ping Li, Hui Chen, Hong-Wei Li

**Affiliations:** ^1^Department of Cardiology, Cardiovascular Center, Beijing Friendship Hospital, Capital Medical University, Beijing, China; ^2^Department of Internal Medicine and Geriatrics, Beijing Friendship Hospital, Capital Medical University, Beijing, China; ^3^Beijing Key Laboratory of Metabolic Disorder Related Cardiovascular Disease, Beijing, China

**Keywords:** pulse pressure, acute coronary syndrome, all-cause mortality, cardiac mortality, cohort study

## Abstract

**Background:**

Pulse pressure (PP) is a surrogate of aortic stiffness, and reflects cardiac performance and stroke volume. Previous studies have indicated that PP was a robust predictor of cardiovascular outcomes and mortality. However, results have been mixed, and very few studies have focused on the association of PP with mortality in acute coronary syndrome (ACS). Thus, we aimed to investigate the relationship between admission PP and the prognosis of patients with ACS.

**Methods:**

This cohort study included 10,824 patients diagnosed with ACS from the Cardiovascular Center Beijing Friendship Hospital Database Bank (CBDBANK) from January 2013 to October 2018. Patients with cardiogenic shock, malignancy, severe trauma and, no PP at admission were excluded. Restricted cubic spline and Cox proportional hazards regression were used to evaluate the association between PP and 1-year all-cause and cardiac mortality.

**Results:**

In the whole cohort, a total of 237 (2.19%) all-cause deaths were reported at 1-year follow-up. Restricted cubic spline analysis suggested a J-shaped relationship between PP and mortality. Among patients with ACS, both lower and higher PP levels were related to an increased risk of mortality (*P*_non–linear_ < 0.001); with a PP level of 30 or 80 mmHg, as compared with 50 mmHg, the adjusted hazard ratios for 1-year all-cause mortality were 2.02 (95% CI, 1.27–3.22) and 1.62 (95% CI, 1.13–2.33), respectively, after adjustments for potential confounders. Similar results were observed for cardiac deaths. The J-shaped relationship between PP and mortality remained in a series of subgroup analyses.

**Conclusion:**

Our results suggested that both low and high PP were associated with an increased risk of mortality in patients with ACS.

## Introduction

Arterial hypertension is one of the well-known risk factors for atherosclerosis (1) and can increase the risk of death in patients with acute coronary syndrome (ACS). Currently, the criteria for diagnosis, treatment, and evaluation of therapeutic efficacy for hypertension are mainly based on the levels of systolic blood pressure (SBP) and/or diastolic blood pressure (DBP). Historically, a linear relationship between increasing SBP and the risk of adverse cardiovascular outcomes has been reported (2, 3). The famous SPRINT (Systolic Blood Pressure Intervention Trial) study conducted at 102 clinical sites in the United States, suggested an aggressive treatment of SBP (<120 mmHg) in high-risk non-diabetic patients (4). However, aggressive reduction of blood pressure, particularly DBP, may impair myocardial perfusion and lead to an adverse outcome, especially in patients with ischemic heart disease.

Pulse pressure (PP) calculated as SBP minus DBP, has been indicated as a more robust prognostic predictor of cardiovascular disease than SBP, DBP, or mean arterial pressure (MAP) by numerous previous population-based studies (5–11). However, results have been mixed, and most of these studies focused on PP measured in non-acute cardiovascular situations. Very few studies have focused on the association of mortality with PP in ACS patients, and the studies that have been performed show inconsistent results (12–17). The majority of these studies (12–15) indicated a substantial prognostic value of low PP in patients with ACS, which may provoke confusion since some other studies (16, 17) have shown that high, rather than low, PP was associated with higher mortality and risk of adverse outcomes. In light of this disparity, this study aimed to further explore the relationship between PP and clinical outcomes across the spectrum of ACS.

## Materials and Methods

### Study Design

This was an observational cohort study. The Cardiovascular Center Beijing Friendship Hospital Database Bank (CBDBANK) collected the medical records of inpatients diagnosed with ACS in the Department of Cardiology of Beijing Friendship Hospital. Patients were under standard medical and interventional management for ACS. Diagnostic criteria for ACS (including ST-segment elevation myocardial infarction [STEMI], non-ST-segment elevation myocardial infarction [NSTEMI], and unstable angina unstable angina pectoris [UAP]) were based on relevant guidelines (18, 19). Acute myocardial infarction (AMI) was defined as a typical rise and/or fall of cardiac troponin values with at least one value above the 99th percentile upper reference limit (URL) and at least one of the following: symptoms of acute myocardial ischemia; new significant ST segment or T wave change or new-onset left bundle branch block; development of pathologic Q wave in ≥2 contiguous electrocardiogram leads; imaging evidence of new loss of viable myocardium or new regional wall motion abnormality in a pattern consistent with an ischemic etiology; and identification of intracoronary lesion by angiography. UAP was defined as myocardial ischemia at rest or on minimal exertion in the absence of acute cardiomyocyte injury/necrosis.

The study was approved by the Ethics Committee of Beijing Friendship Hospital, Capital Medical University and performed according to the guidelines of the Helsinki Declaration.

### Population

**FIGURE 1:**
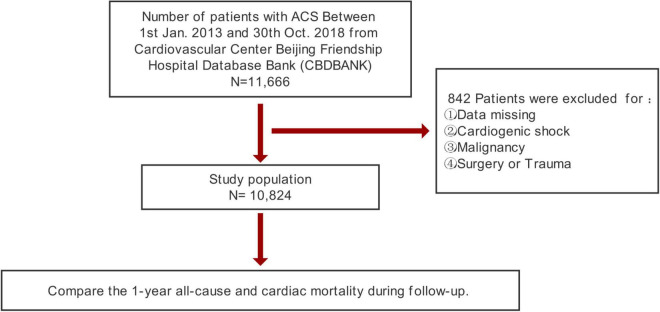
Flowchart of study population selection.

A total of 11,666 inpatients diagnosed with ACS were collected in CBDBANK from January 2013 to October 2018. Patients with cardiogenic shock (clinical criteria: SBP < 90 mmHg for ≥30 min or catecholamines to maintain SBP > 90 mmHg, and clinical pulmonary congestion and impaired end-organ perfusion [altered mental status, cold/clammy skin and extremities, urine output <30 ml/h, or lactate >2.0 mmol/L], or a class IV rating according to the Killip classification) (20, 21), malignancy, and severe trauma; patients who recently underwent surgical operations; and patients with no PP at admission were excluded. Overall, 10,824 patients were included in this study (Figure 1).

### Measurement of Blood Pressure

Blood pressure was measured by cardiologists using the validated upper arm medical electronic (OMRON HBP-1300 blood pressure monitor) or mercury sphygmomanometer immediately when patients were admitted to the Cardiology Department of Beijing Friendship Hospital. Those who had STEMI were measured after reperfusion. All patients lay down for at least 5 minutes in a quiet room before the blood pressure measurement, and the patient’s upper arms were kept at the heart level. Measurements on both arms were taken, and the arm with a higher value was used as the reference. PP was defined as SBP minus DBP.

### Covariates

Demographic characteristics, medical history, laboratory test results, diagnoses, and medical therapy were collected by a standardized procedure. Based on prior knowledge, the following covariates were collected including: age, gender, body mass index (BMI), heart rate, medical history (hypertension, diabetes mellitus, myocardial infarction, chronic kidney disease [CKD], stroke, dyslipidemia, chronic heart failure, and smoking), medication used before admission (dual-antiplatelet therapy [DAPT], angiotensin-converting enzyme inhibitor [ACEI] or angiotensin receptor blocker [ARB], beta-blocker, and statins), laboratory results (fasting plasma glucose [FPG], glycosylated hemoglobin [HbA1c], total cholesterol, triglyceride, low-density lipoprotein cholesterol [LDL-C], high-density lipoprotein cholesterol, high sensitivity C-reactive protein [hs-CRP], peak value of cardiac troponin I [cTnI], peak value of N-terminal pro-brain natriuretic peptide [NT-proBNP], and estimated glomerular filtration rate [eGFR]), Killip class, diagnosis of atrial fibrillation, angiography findings (left main coronary artery lesion, multi-vessel lesion, chronic total occlusion lesion), and in-hospital treatments (percutaneous coronary intervention [PCI], DAPT, ACEI/ARB, beta-blocker and statins). Medical history was identified according to the self-reported history of diagnosis. Overnight fasting blood samples were obtained and tested in a central laboratory by standard methods. eGFR was calculated using the MDRD (The Modification of Diet in Renal Disease) formula: [eGFR (ml/min/1.73 m^2^) = 175 × (serum creatinine)^–1.154^ × (Age)^–0.203^ × (0.742 if female) × (1.212 if African American)] (22). Echocardiograms were performed by expert cardiologists or certified sonographers. Left ventricular ejection fraction (LVEF) was assessed with echocardiography using the Simpsons method, with LVEF ≤ 50% indicating left ventricular systolic dysfunction. The coronary angiography and PCI operation were performed according to the standard techniques by experienced cardiologists.

### Study Outcomes

Patients’ outcomes during hospitalization were collected and confirmed based on their medical records. Follow-up information, particularly vital signs (and date of death whenever applicable) was collected for each participant at 1, 3, and 6 months, and every year after discharge until death. This information was obtained by phone calls or from medical records if patients came to the outpatient clinic for a follow-up examination. The primary endpoint was 1-year all-cause mortality during follow-up. 1-year cardiac mortality was a secondary outcome. Cardiac death was defined as death caused by AMI, heart failure, or documented sudden cardiac death.

### Statistical Analysis

Clinical characteristics were presented as proportions, mean (standard deviation) or median (interquartile range). Baseline characteristics were summarized according to vital status at 1-year follow-up and compared between survivors and decedents using the χ^2^ test, *t*-test, or Mann–Whitney U test, as appropriate.

Cox proportional hazards regression models were used to calculate the adjusted hazard ratios (HRs) and 95% confidence intervals (CIs) for PP and 1-year mortality. Our primary analysis was based on restricted cubic splines to facilitate detailed description of the dose-response curves between PP and all-cause mortality as well as cardiac mortality (23). Using spline regression, we calculated HRs and 95% CIs for specific admission PP values and depicted the shape of the overall dose-response, and tested for linear and non-linear shapes of each association. Restricted cubic splines were fitted with 4 knots placed at the 5th, 35th, 65th, and 95th percentiles across the range of PP. The statistical significance (at the 0.05 level) of the overall association and the non-linearity of the risk curves was evaluated with Wald tests (23, 24). We enrolled the following confounders known to influence the prognosis of ACS in the spline Cox models: age, gender, BMI, previous diabetes mellitus, previous hypertension, previous myocardial infarction, current smoking, atrial fibrillation, previous DAPT, heart rate, LVEF < 50%, HbA1c, LDL-C, eGFR, the peak value of cTnI, angiography findings (left main coronary artery lesion, multi-vessel lesion, and chronic total occlusion lesion), and in-hospital treatments (PCI, DAPT, ACEI/ARB, and statins). Adjusted survival curves were calculated based on the final spline Cox model for 1-year all-cause and cardiac mortality at specific PP values (25).

In subgroup analyses, we examined results with separate splines in accordance with ACS types (UAP, AMI); age (<65 years, ≥65 years); gender (male, female); BMI (<25 kg/m^2^, ≥25 kg/m^2^); previous hypertension (with; without); previous diabetes mellitus (with; without); eGFR (<60 ml/min/1.73m^2^, ≥60 ml/min/1.73m^2^); and smoking status (current smoker; non-smoker).

A *p*-value < 0.05 was considered statistically significant. All data analyses were carried out using the Stata software, version 17.0 (StataCorp LP, College Station, TX, United States), and R software, version 4.1.2 (R Foundation for Statistical Computing).

## Results

The current study included 10,824 patients (Figure 1). Table 1 presents the summary of the baseline clinical characteristics and the laboratory test results according to vital status at 1-year follow-up. The PP levels were significantly higher in decedents than those in survivors. Decedents also tended to be older, with lower levels of BMI, with higher prevalence of previous diabetes, myocardial infarction, CKD, and chronic heart failure, and with higher levels of heart rate, FPG, HbA1c, hs-CRP, the peak value of cTnI, and peak value of NT-proBNP. In addition, decedents were prone to have lower levels of eGFR and LVEF. Notably, decedents showed a lower rate of receiving PCI, DAPT, ACEI/ARB, beta-blockers, and statins during hospitalization.

**TABLE 1:** Baseline characteristics by vital status at 1-year follow-up.

	Total population (n = 10,824)	Survivors (*n* = 10,587)	Decedents (*n* = 237)	*P-value*
**Age, years**	65.2 (10.9)	65.0 (10.8)	73.5 (11.2)	<0.001
**Male, No. (%)**	6899 (63.7)	6752 (63.8)	147 (62.0)	0.58
**BMI, kg/m^2^**	25.8 (3.6)	25.8 (3.6)	23.9 (4.1)	<0.001
**SBP, mmHg**	131.6 (18.8)	131.6 (18.6)	135.2 (24.4)	0.003
**DBP, mmHg**	75.5 (11.3)	75.6 (11.2)	75.0 (12.9)	0.48
**PP, mmHg**	56.1 (15.8)	56.0 (15.6)	60.2 (21.3)	<0.001
**Heart rate, bpm**	71.7 (12.9)	71.5 (12.7)	79.7 (18.2)	<0.001
**Medical history, No. (%)**				
**Hypertension**	7656 (70.7)	7476 (70.6)	180 (75.9)	0.074
**Diabetes mellitus**	3809 (35.2)	3710 (35.0)	99 (41.8)	0.032
**Myocardial infarction**	992 (9.2)	947 (8.9)	45 (19.0)	<0.001
**Chronic kidney disease**	433 (4.0)	402 (3.8)	31 (13.1)	<0.001
**Stroke**	1893 (17.5)	1843 (17.4)	50 (21.1)	0.14
**Dyslipidemia**	4852 (44.8)	4766 (45.0)	86 (36.3)	0.008
**Chronic heart failure**	73 (0.7)	65 (0.6)	8 (3.4)	<0.001
**Smoking**	3693 (34.1)	3630 (34.3)	63 (26.6)	0.013
**Medication used before admission, No. (%)**				
**DAPT**	1708 (15.8)	1656 (15.6)	52 (21.9)	0.009
**ACEI/ARB**	3785 (35.0)	3708 (35.0)	77 (32.5)	0.42
**Beta-blocker**	2546 (23.5)	2496 (23.6)	50 (21.1)	0.37
**Statins**	2887 (26.7)	2842 (26.8)	45 (19.0)	0.007
**Laboratory values**				
**FPG, mmol/L**	6.1 (2.2)	6.1 (2.2)	6.9 (3.3)	<0.001
**HbA1c,%**	6.4 (1.3)	6.4 (1.3)	6.7 (1.5)	<0.001
**TC, mmol/L**	4.3 (1.0)	4.3 (1.0)	4.2 (1.2)	0.089
**TG, mmol/L**	1.4 (1.0, 2.0)	1.4 (1.0, 2.0)	1.3 (0.9, 1.5)	<0.001
**LDL-C, mmol/L**	2.4 (0.8)	2.4 (0.8)	2.4 (0.9)	0.40
**HDL-C, mmol/L**	1.1 (0.3)	1.1 (0.3)	1.1 (0.3)	0.018
**hs-CRP, mg/L**	2.0 (0.8, 6.1)	2.0 (0.8, 5.9)	5.5 (2.0, 17.2)	<0.001
**LVEF,%**	64.0 (9.3)	64.2 (9.1)	54.4 (13.0)	<0.001
**Peak value of cTnI, ng/mL**	0.0 (0.0, 0.1)	0.0 (0.0, 0.1)	0.1 (0.0, 1.4)	<0.001
**Peak value of NT-proBNP, pg/mL**	287 (119, 844)	287 (116, 770)	4346 (785, 16066)	<0.001
**eGFR, ml/min/1.73m^2^**	80.3 (21.4)	80.8 (21.0)	59.9 (25.5)	<0.001
**Initial diagnosis, No. (%)**				<0.001
**UAP**	7705 (71.2)	7626 (72.0)	79 (33.3)	
**AMI**	3119 (28.8)	2961 (28.0)	158 (66.7)	
**NSTEMI**	1611 (14.9)	1520 (14.4)	91 (38.4)	
**STEMI**	1508 (13.9)	1441 (13.6)	67 (28.3)	
**Killip class, No. (%)**				<0.001
**I**	5190 (51.5)	5125 (52.1)	65 (28.5)	
**II**	4230 (42.0)	4113 (41.8)	117 (51.3)	
**III**	651 (6.5)	605 (6.1)	46 (20.2)	
**Atrial fibrillation**	671 (6.2)	624 (5.9)	47 (19.8)	<0.001
**Angiography findings, No. (%)**				
**LM lesion**	940 (8.7)	915 (8.6)	25 (10.5)	0.30
**Multi-vessel lesion**	7130 (65.9)	7026 (66.4)	104 (43.9)	<0.001
**Chronic total occlusion lesion**	2291 (21.2)	2238 (21.1)	53 (22.4)	0.65
**In-hospital treatments, No. (%)**				
**PCI**	5567 (51.4)	5490 (51.9)	77 (32.5)	<0.001
**DAPT**	5949 (55.0)	5834 (55.1)	115 (48.5)	0.044
**ACEI/ARB**	6034 (55.7)	5926 (56.0)	108 (45.6)	0.001
**Beta-blocker**	7464 (69.0)	7330 (69.2)	134 (56.5)	<0.001
**Statins**	9535 (88.1)	9371 (88.5)	164 (69.2)	<0.001

*Values are presented as mean (SD), median (IQR) or number (%).*

*ACEI, angiotensin-converting enzyme inhibitor; ARB, angiotensin receptor blocker; AMI, acute myocardial infarction; BMI, body mass index; cTnI, cardiac troponin I; CCB, calcium channel blocker; DAPT, dual-antiplatelet therapy; DBP, diastolic blood pressure; eGFR, estimated glomerular filtration rate; FPG, fasting plasma glucose; HbA1c, glycosylated hemoglobin; HDL-C, high-density lipoprotein cholesterol; hs-CRP, high sensitivity C-reactive protein; LDL-C, low-density lipoprotein cholesterol; LM, left main coronary artery; LVEF, left ventricular ejection fraction; NT-proBNP, N-terminal pro-brain natriuretic peptide; NSTEM, non-ST-elevation myocardial infarction; PCI, percutaneous coronary intervention; PP, pulse pressure; SD, standard deviation; SBP, systolic blood pressure; STEMI, ST-elevation myocardial infarction; TC, total cholesterol; TG, triglyceride; UAP, unstable angina pectoris.*

**FIGURE 2:**
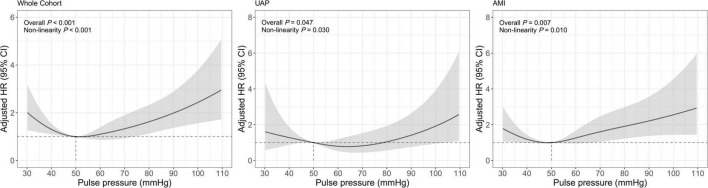
Restricted cubic spline analysis for association of PP and 1-year all-cause mortality. Adjusted model included age, gender, BMI, previous diabetes mellitus, previous hypertension, previous MI, current smoking, atrial fibrillation, previous DAPT, heart rate, LVEF < 50%, HbA1c, LDL-C, eGFR, peak value of cTnI, angiography findings (LM lesion, multi-vessel lesion, chronic total occlusion lesion), and in-hospital treatments (PCI, DAPT, ACEI/ARB, statins).

A total of 237 (2.19%) all-cause deaths were reported, including 79 (1.03%) of the 7,705 UAP patients and 158 (5.07%) of the 3,119 AMI patients during 1-year follow-up. Associations between PP levels and 1-year all-cause mortality were shown in Table 2 and Figure 2. The multivariable-adjusted spline Cox models were shown in Supplementary Table 1. In the whole cohort, the risk of all-cause mortality increased with both lower or higher PP levels (*P*_non–linear_ < 0.001), which indicated a J-shaped relationship between PP and all-cause mortality. For a PP level of 30 mmHg, as compared with 50 mmHg, the adjusted HR for 1-year all-cause mortality was 2.02 (95% CI, 1.27–3.22). While, for a PP level of 80 mmHg, the adjusted HR for 1-year all-cause mortality was 1.62 (95% CI, 1.13–2.33). Moreover, among AMI patients, lower and higher PP levels were also associated with increased 1-year all-cause mortality (*P*_non–linear_ = 0.010). The adjusted HRs for a PP level of 30 mmHg and 80 mmHg were 1.80 (95% CI, 1.07–3.03) and 1.91 (95% CI, 1.22–2.98), respectively.

**TABLE 2:** Risk of 1-year all-cause mortality associated with PP levels in whole cohort, UAP patients, and AMI patients.

PP levels	Hazard ratio (95% CI)*		
	
	Whole cohort	UAP	AMI
**30 mmHg**	2.02 (1.27–3.22)	1.61 (0.59–4.41)	1.80 (1.07–3.03)
**40 mmHg**	1.30 (1.10–1.55)	1.28 (0.88–1.87)	1.19 (0.98–1.43)
**50 mmHg**	1.00 [reference]	1.00 [reference]	1.00 [reference]
**60 mmHg**	1.10 (0.87–1.39)	0.80 (0.53–1.20)	1.26 (0.95–1.67)
**70 mmHg**	1.33 (0.93–1.90)	0.82 (0.44–1.53)	1.61 (1.05–2.46)
**80 mmHg**	1.62 (1.13–2.33)	1.04 (0.56–1.93)	1.91 (1.22–2.98)
**90 mmHg**	1.99 (1.37–2.89)	1.41 (0.76–2.64)	2.21 (1.37–3.56)
**100 mmHg**	2.43 (1.56–3.79)	1.92 (0.94–3.93)	2.55 (1.44–4.54)
***P* for Non-linearity**	<0.001	0.030	0.010

*Estimates were adjusted for age, gender, BMI, previous diabetes mellitus, previous hypertension, previous MI, current smoking, atrial fibrillation, previous DAPT, heart rate, LVEF < 50%, HbA1c, LDL-C, eGFR, peak value of cTnI, angiography findings (LM lesion, multi-vessel lesion, and chronic total occlusion lesion), and in-hospital treatments (PCI, DAPT, ACEI/ARB, and statins).

*Abbreviations see Table 1.*

**FIGURE 3:**
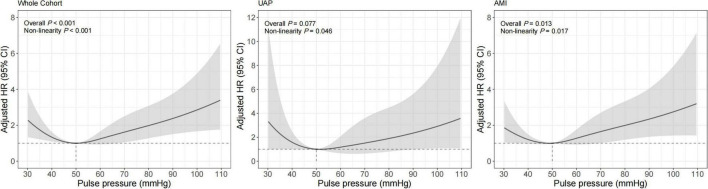
Restricted cubic spline analysis for association of PP and 1-year cardiac mortality. Adjusted model included age, gender, BMI, previous diabetes mellitus, previous hypertension, previous MI, current smoking, atrial fibrillation, previous DAPT, heart rate, LVEF < 50%, HbA1c, LDL-C, eGFR, peak value of cTnI, angiography findings (LM lesion, multi-vessel lesion, chronic total occlusion lesion), and in-hospital treatments (PCI, DAPT, ACEI/ARB, statins).

Similar results were observed for the association between PP and 1-year cardiac mortality (Table 3 and Figure 3). The multivariable-adjusted spline Cox models were shown in Supplementary Table 2. In the whole cohort, the risk of cardiac mortality was higher in both lower or higher PP levels (*P*_non–linear_ < 0.001). For a PP level of 30 mmHg, as compared with 50 mmHg, the adjusted HR for 1-year cardiac mortality was 2.30 (95% CI, 1.34–3.93). While, for a PP level of 80 mmHg, the adjusted HR for 1-year cardiac mortality was 1.98 (95% CI, 1.28–3.08). A similar relationship between PP and cardiac mortality (*P*_non–linear_ = 0.017) was observed in AMI patients that the adjusted HRs for a PP level of 30 mmHg and 80mmHg were 1.87 (95% CI, 1.03–3.39) and 1.99 (95% CI, 1.19–3.33), respectively. However, among UAP patients, PP was no longer significantly associated with 1-year all-cause or cardiac mortality, although patients with higher PP showed a trend toward increased risk for mortality.

**TABLE 3:** Risk of 1-year cardiac mortality associated with PP levels in whole cohort, UAP patients, and AMI patients.

PP levels	Hazard Ratio (95% CI)*		
	
	Whole Cohort	UAP	AMI
**30 mmHg**	2.30 (1.34–3.93)	3.35 (0.98–11.44)	1.87 (1.03–3.39)
**40 mmHg**	1.34 (1.10–1.63)	1.58 (1.00–2.48)	1.20 (0.97–1.49)
**50 mmHg**	1.00 [reference]	1.00 [reference]	1.00 [reference]
**60 mmHg**	1.24 (0.93–1.66)	1.17 (0.66–2.08)	1.28 (0.92–1.76)
**70 mmHg**	1.62 (1.05–2.50)	1.51 (0.63–3.60)	1.65 (1.01–2.70)
**80 mmHg**	1.98 (1.28–3.08)	1.89 (0.80–4.48)	1.99 (1.19–3.33)
**90 mmHg**	2.38 (1.50–3.76)	2.35 (0.98–5.62)	2.34 (1.35–4.05)
**100 mmHg**	2.85 (1.66–4.89)	2.91 (1.07–7.94)	2.75 (1.43–5.29)
***P* for Non-linearity**	<0.001	0.046	0.017

*Estimates were adjusted for age, gender, BMI, previous diabetes mellitus, previous hypertension, previous MI, current smoking, atrial fibrillation, previous DAPT, heart rate, LVEF < 50%, HbA1c, LDL-C, eGFR, peak value of cTnI, angiography findings (LM lesion, multi-vessel lesion, and chronic total occlusion lesion), and in-hospital treatments (PCI, DAPT, ACEI/ARB, and statins).

*Abbreviations see Table 1.*

**FIGURE 4:**
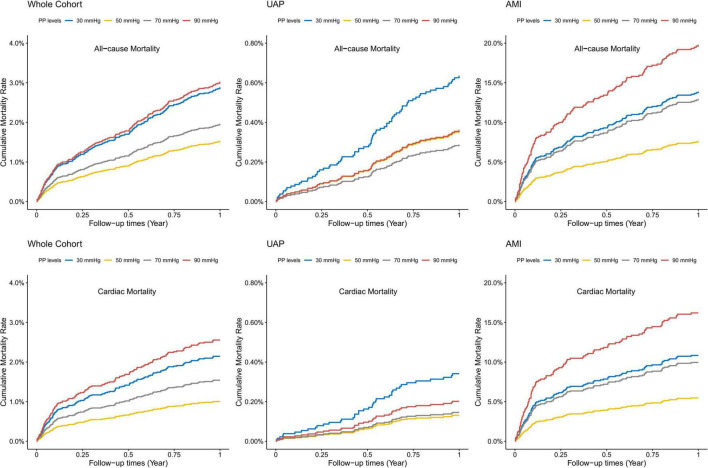
Adjusted survival curves for 1-year all-cause and cardiac mortality according to different PP levels. Adjusted model included age, gender, BMI, previous diabetes mellitus, previous hypertension, previous MI, current smoking, atrial fibrillation, previous DAPT, heart rate, LVEF < 50%, HbA1c, LDL-C, eGFR, peak value of cTnI, angiography findings (LM lesion, multi-vessel lesion, chronic total occlusion lesion), and in-hospital treatments (PCI, DAPT, ACEI/ARB, statins).

**FIGURE 5:**
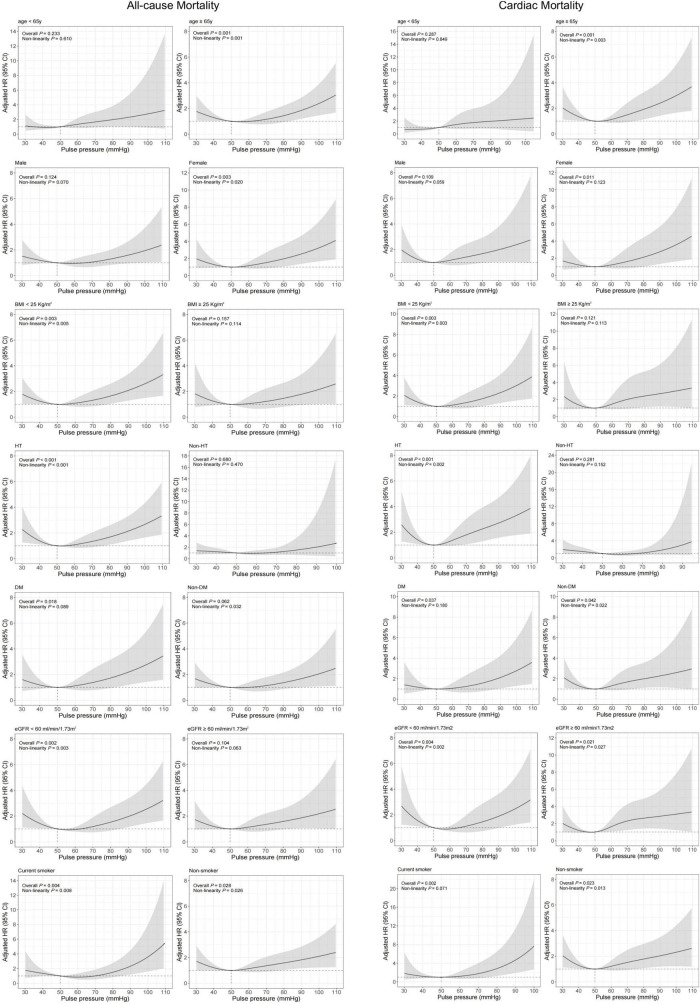
Restricted cubic spline analysis for association of PP and 1-year all-cause and cardiac mortality according to different subgroups. Adjusted model included age, gender, BMI, previous diabetes mellitus, previous hypertension, previous MI, current smoking, atrial fibrillation, previous DAPT, heart rate, LVEF < 50%, HbA1c, LDL-C, eGFR, peak value of cTnI, angiography findings (LM lesion, multi-vessel lesion, chronic total occlusion lesion), and in-hospital treatments (PCI, DAPT, ACEI/ARB, statins).

Covariates-adjusted survival curves of time until all-cause and cardiac mortality were shown in Figure 4. Patients with both lower and higher levels of PP were associated with a higher mortality rate at 1-year follow-up. Subgroup analyses were performed for all-cause and cardiac mortality outcomes by the following variables: age, gender, BMI, previous hypertension, previous diabetes mellitus, eGFR, and smoking status (Figure 5). The J-shaped relationship between PP and all-cause mortality was more pronounced among patients aged ≥65 years, female, patients with BMI < 25 kg/m^2^, patients with previous hypertension, patients without previous diabetes, patients with eGFR < 60 ml/min/1.73m^2^, and both current smokers and non-smokers. The results did not significantly change between PP and cardiac mortality.

## Discussion

In this study of patients across the spectrum of ACS, a J-shaped relationship between PP and 1-year all-cause and cardiac mortality was observed with increased mortality in patients with lower and higher PP levels. Subgroup analyses confirmed the significance of the relationship in patients with AMI, which was attenuated in patients with UAP.

The robust cardiovascular prognostic value of PP has been identified in numerous population-based studies (5–10, 26–28). One study including 2,152 individuals aged over 65 years showed that higher PP was an independent predictor of incident coronary heart disease and heart failure among the community elderly (8). In the PROCAM study of 5,389 men aged 35–65, increased PP was shown to be an important independent determinant of coronary risk (10). The REasons for Geographic And Racial Differences in Stroke (REGARDS) study found that PP was an independent risk factor for acute coronary heart disease in the national cohort of 30,239 black and white participants aged over 45 years old (28). However, these findings were discovered in non-acute cardiovascular situations. Investigations of the association of PP with the outcome within one year in patients with ACS are scarce and somewhat contradictory. Here for the first time, we provide evidence from a population-based real-world cohort of ACS and use PP as a continuous variable to reveal the association between PP and the prognosis of ACS using spline regression.

We postulated that higher PP may be associated with worse outcomes, since it had been reported in multiple non-acute situation studies and also some ACS studies (16, 17). However, our study also showed a strong association between lower PP and worse prognosis in patients with ACS, which indicated a J-shaped relationship between PP and mortality. The J-shaped relationship was defined as an increase in adverse cardiovascular outcomes below or above a certain blood pressure, with the trough representing the point that had the lowest risk of adverse events (29). This phenomenon has also been mentioned in studies of ACS patients. In a five-year follow-up of 353 ACS patients aged at least 80 years, the mortality rate increased in patients with PP ≤ 50 mmHg or >70 mmHg (13). A more recent study by Gjin Ndrepepa et al. (30) suggested a U-shaped relationship between central PP and 8-year all-cause or cardiac mortality in patients with STEMI undergoing PCI. However, after adjustment of demographic and clinical variables such as infarct size, postprocedural TIMI flow grade, age, sex, diabetes, smoking, and hypercholesterolemia, the association between central PP and the long-term prognosis was attenuated. In contrast, the association remained after adjustment for potential confounders in our study, which indicated PP as an independent prognostic predictor.

The mechanism underlying the J-shaped phenomenon was unclear and debated, especially for the explanation for the adverse prognosis of lower PP. Warren J. et al. (31), on the other hand, suggested that a lower PP in the setting of PCI independently predicted lower long-term mortality. This study emphasized the protective role of high DBP in coronary perfusion for a long-term prognosis in patients undergoing PCI. Whereas, there was no difference between PP groups in the rate of cardiac and all-cause mortality at 1-year post-procedure. Conversely, a previous study by El-Menyar et al. (12) has shown that lower PP was an independent predictor of stroke and in-hospital mortality in overall ACS. A multicenter study involving 14,514 ACS patients showed that PP < 49 mmHg was independently associated higher in-hospital mortality compared with PP > 76 mmHg (OR = 2.57, 95% CI, 1.80–3.67) (14). In fact, PP is not only associated with the degree of aortic stiffness but also the ventricular stroke volume (32). When the patient’s stroke volume decreases (as a decrease in LVEF in clinical), PP is no longer the marker of arteriosclerosis (33). One study of the SPUM-ACS cohort indicated that the J-shaped relationship disappeared after the exclusion of patients with LVEF ≤ 40%, indicating that LVEF is a very powerful confounder that has specific statistical interaction with mortality (16). In the present study, as was shown in the Kaplan-Meier survival curve according to PP categories in patients with PP < 50 mmHg (Supplementary Table 3 and Supplementary Figure 1), patients with SBP < 120 mmHg were associated with higher mortality regardless of DBP. Thus, from our perspective, lower PP might help identify patients with a high risk of mortality because of reduced cardiac functional reserve. In addition, in our subgroup analyses, the J-shaped relationship was more obvious in patients with a diagnosis of AMI, aged ≥65 years, previous hypertension, and eGFR < 60 ml/min/1.73m^2^. The adverse prognosis of lower PP might be affected by the severity of the disease and chronic condition.

The mechanism underlying the relationship between PP and mortality remains unclear. Physiologically, there is a linear rise in both SBP and DBP until age 60 after which SBP continues to rise and DBP begins to decrease. The subsequent increase in PP is consistent with the increased large artery stiffness (34). Blood pressure is a complicated variable but can be broken down into two components: the steady-state (e.g., MAP) and the pulsatile (e.g., PP). PP is determined by stroke volume, wave reflections and arterial stiffness (35). Aging and diseases (e.g., hypertension, CKD, diabetes, and atherosclerosis) could cause structural changes in blood vessels, exacerbating arterial stiffness. Fragmentation of elastin and increased deposition of collagen change the patient’s collagen to elastin ratio and cause arterial calcification. Glycation of both elastin and collagen fibers and crosslinking of collagen molecules by advanced glycation end-products can compromise vessel compliance (36–38). The loss of elastic recoil and earlier timing of reflected pressure waves in late systole reduce the DBP. Therefore, the “de-stiffening” strategy based on the antihypertrophic effect on vascular smooth muscle cells by blocking angiotensin and/or aldosterone system is proposed to reduce cardiovascular risk (39). It is possible to selectively reduce central SBP and PP by decreasing arterial stiffness and/or wave reflections. In most studies focusing on therapeutic efficacy, the renin-angiotensin system inhibition combined with diuretics or calcium antagonists (40, 41) were recommended, whereas the renin-angiotensin system inhibition combined with classic beta-blockers was not, because the beta-blockers are shown to have little or no effect on central PP (42). Because cardiovascular risk reduction is mainly associated with the management of central SBP and PP, this strategy should be extensively evaluated in the future.

Our study had several limitations. First, despite the fact that all data in this study were collected prospectively, our study was limited by its retrospective design. Second, the long-term monitoring of blood pressure was insufficient, and admission PP only partially reflected the status of patients’ blood pressure management. Third, the severity of myocardial ischemia or infarct size was not included in this research. Finally, the underlying mechanisms for the association between adverse outcomes and lower PP were not completely clear, and further investigations were needed in the future.

## Conclusion

In summary, PP is an independent prognostic factor for 1-year all-cause and cardiac mortality in patients with ACS. The present study demonstrates a J-shaped relationship between PP and the prognosis of ACS, especially in patients with AMI. Further prospective cohort studies are needed to confirm these findings and elucidate the underlying mechanisms.

## Data Availability Statement

The raw data supporting the conclusions of this article will be made available by the authors, without undue reservation.

## Ethics Statement

The studies involving human participants were reviewed and approved by the Institutional Review Board of Beijing Friendship Hospital affiliated to Capital Medical University. The patients/participants provided their written informed consent to participate in this study.

## Author Contributions

H-WL designed the protocol of the study. MW drafted the manuscript. MW, WS, C-YJ, W-PL, HC, and H-WL participated in the collection, interpretation, and analysis of the data. All authors approved the final version for publication.

## Conflict of Interest

The authors declare that the research was conducted in the absence of any commercial or financial relationships that could be construed as a potential conflict of interest.

## Publisher’s Note

All claims expressed in this article are solely those of the authors and do not necessarily represent those of their affiliated organizations, or those of the publisher, the editors and the reviewers. Any product that may be evaluated in this article, or claim that may be made by its manufacturer, is not guaranteed or endorsed by the publisher.

## References

[1] Dzau VJ. Atherosclerosis and hypertension: mechanisms and Interrelationships. *J Cardiovasc Pharmacol.* (1990) 15(Suppl. 5):S59–64. 1694933

[2] Lewington S Clarke R Qizilbash N Peto R Collins R. Age-specific relevance of usual blood pressure to vascular mortality: a meta-analysis of individual data for one million adults in 61 prospective studies. *Lancet.* (2002) 360:1903–13. 10.1016/s0140-6736(02)11911-8 12493255

[3] Staessen JA Gasowski J Wang JG Thijs L Den Hond E Boissel JP Risks of untreated and treated isolated systolic hypertension in the elderly: meta-analysis of outcome trials. *Lancet.* (2000) 355:865–72. 10.1016/s0140-6736(99)07330-4 10752701

[4] Wright JT Jr Williamson JD Whelton PK Snyder JK Sink KM Rocco MV A randomized trial of intensive versus standard blood-pressure control. *N Engl J Med.* (2015) 373:2103–16. 10.1056/NEJMoa1511939 26551272 PMC4689591

[5] Millar JA Lever AF Burke V. Pulse pressure as a risk factor for cardiovascular events in the MRC mild hypertension trial. *J Hypertens.* (1999) 17:1065–72. 10.1097/00004872-199917080-00004 10466460

[6] Benetos A Rudnichi A Safar M Guize L. Pulse pressure and cardiovascular mortality in normotensive and hypertensive subjects. *Hypertension.* (1998) 32:560–4. 10.1161/01.hyp.32.3.560 9740626

[7] Franklin SS Khan SA Wong ND Larson MG Levy D. Is pulse pressure useful in predicting risk for coronary heart disease? The Framingham heart study. *Circulation.* (1999) 100:354–60. 10.1161/01.cir.100.4.354 10421594

[8] Vaccarino V Holford TR Krumholz HM. Pulse pressure and risk for myocardial infarction and heart failure in the elderly. *J Am Coll Cardiol.* (2000) 36:130–8. 10.1016/s0735-1097(00)00687-2 10898424

[9] Sesso HD Stampfer MJ Rosner B Hennekens CH Gaziano JM Manson JE Systolic and diastolic blood pressure, pulse pressure, and mean arterial pressure as predictors of cardiovascular disease risk in men. *Hypertension.* (2000) 36:801–7. 10.1161/01.hyp.36.5.801 11082146

[10] Assmann G Cullen P Evers T Petzinna D Schulte H. Importance of arterial pulse pressure as a predictor of coronary heart disease risk in PROCAM. *Eur Heart J.* (2005) 26:2120–6. 10.1093/eurheartj/ehi467 16141262

[11] Petrie CJ Voors AA Robertson M van Veldhuisen DJ Dargie HJA. Low pulse pressure predicts mortality in subjects with heart failure after an acute myocardial infarction: a post-hoc analysis of the Capricorn study. *Clin Res Cardiol.* (2012) 101:29–35. 10.1007/s00392-011-0360-x 21935656

[12] El-Menyar A Zubaid M Almahmeed W Alanbaei M Rashed W Al Qahtani A Initial hospital pulse pressure and cardiovascular outcomes in acute coronary syndrome. *Arch Cardiovasc Dis.* (2011) 104:435–43. 10.1016/j.acvd.2011.05.008 21944145

[13] Li S Barywani S Fu M. Prognostic power of lower pulse pressure on long-term all-cause mortality in octogenarians with acute coronary syndrome: a propensity-score-matched cohort study. *J Hypertens.* (2015) 33:279–86. 10.1097/hjh.0000000000000403 25318655

[14] Tan NS Sarak B Fox KA Brieger D Steg PG Gale CP Pulse pressure in acute coronary syndromes: comparative prognostic significance with systolic blood pressure. *Eur Heart J Acute Cardiovasc Care.* (2019) 8:309–17. 10.1177/2048872617700871 28357882

[15] Ma WF Liang Y Zhu J Yang YM Tan HQ Yu LT Comparison of 4 admission blood pressure indexes for predicting 30-day mortality in patients with st-segment elevation myocardial infarction. *Am J Hypertens.* (2016) 29:332–9. 10.1093/ajh/hpv109 26158853

[16] Harbaoui B Nanchen D Lantelme P Gencer B Heg D Klingenberg R Prognostic value of pulse pressure after an acute coronary syndrome. *Atherosclerosis.* (2018) 277:219–26. 10.1016/j.atherosclerosis.2018.07.013 30033338

[17] Avanzini F Alli C Boccanelli A Chieffo C Franzosi MG Geraci E High pulse pressure and low mean arterial pressure: two predictors of death after a myocardial infarction. *J Hypertens.* (2006) 24:2377–85. 10.1097/01.hjh.0000251897.40002.bf 17082719

[18] Collet J-P Thiele H Barbato E Barthélémy O Bauersachs J Bhatt DL 2020 ESC guidelines for the management of acute coronary syndromes in patients presenting without persistent St-segment elevation: the task force for the management of acute coronary syndromes in patients presenting without persistent St-segment elevation of the European society of cardiology (ESC). *Eur Heart J.* (2020) 42:1289–367. 10.1093/eurheartj/ehaa575 32860058

[19] Thygesen K Alpert JS Jaffe AS Chaitman BR Bax JJ Morrow DA Fourth universal definition of myocardial infarction (2018). *Eur Heart J.* (2018) 40:237–69. 10.1093/eurheartj/ehy462 30165617

[20] Thiele H Zeymer U Neumann FJ Ferenc M Olbrich HG Hausleiter J Intraaortic balloon support for myocardial infarction with cardiogenic shock. *N Engl J Med.* (2012) 367:1287–96. 10.1056/NEJMoa1208410 22920912

[21] Killip T III Kimball JT. Treatment of myocardial infarction in a coronary care unit. A two year experience with 250 patients. *Am J Cardiol.* (1967) 20:457–64. 10.1016/0002-9149(67)90023-9 6059183

[22] Levey AS Coresh J Greene T Stevens LA Zhang YL Hendriksen S Using standardized serum creatinine values in the modification of diet in renal disease study equation for estimating glomerular filtration rate. *Ann Intern Med.* (2006) 145:247–54. 10.7326/0003-4819-145-4-200608150-00004 16908915

[23] Desquilbet L Mariotti F. Dose-response analyses using restricted cubic spline functions in public health research. *Stat Med.* (2010) 29:1037–57. 10.1002/sim.3841 20087875

[24] Harrell FE Jr Lee KL Pollock BG. Regression models in clinical studies: determining relationships between predictors and response. *J Natl Cancer Inst.* (1988) 80:1198–202. 10.1093/jnci/80.15.1198 3047407

[25] Nieto FJ Coresh J. Adjusting survival curves for confounders: a review and a new method. *Am J Epidemiol.* (1996) 143:1059–68. 10.1093/oxfordjournals.aje.a008670 8629613

[26] Madhavan S Ooi WL Cohen H Alderman MH. Relation of pulse pressure and blood pressure reduction to the incidence of myocardial infarction. *Hypertension.* (1994) 23:395–401. 10.1161/01.hyp.23.3.395 8125567

[27] Fang J Madhavan S Cohen H Alderman MH. Measures of blood pressure and myocardial infarction in treated hypertensive patients. *J Hypertens.* (1995) 13:413–9. 7629401

[28] Glasser SP Halberg DL Sands C Gamboa CM Muntner P Safford M. Is pulse pressure an independent risk factor for incident acute coronary heart disease events? The regards study. *Am J Hypertens.* (2014) 27:555–63. 10.1093/ajh/hpt168 24029164 PMC4014855

[29] Rahman F McEvoy JW. The J-shaped curve for blood pressure and cardiovascular disease risk: historical context and recent updates. *Curr Atheroscler Rep.* (2017) 19:34. 10.1007/s11883-017-0670-1 28612327

[30] Ndrepepa G Cassese S Kufner S Xhepa E Fusaro M Laugwitz KL U-shaped association of central pulse pressure with long-term prognosis after St-segment elevation myocardial infarction. *Heart Vessels.* (2019) 34:1104–12. 10.1007/s00380-019-01344-x 30671640

[31] Warren J Nanayakkara S Andrianopoulos N Brennan A Dinh D Yudi M Impact of pre-procedural blood pressure on long-term outcomes following percutaneous coronary intervention. *J Am Coll Cardiol.* (2019) 73:2846–55. 10.1016/j.jacc.2019.03.493 31171090

[32] Dart AM Kingwell BA. Pulse pressure–a review of mechanisms and clinical relevance. *J Am Coll Cardiol.* (2001) 37:975–84. 10.1016/s0735-1097(01)01108-1 11263624

[33] Regnault V Lagrange J Pizard A Safar ME Fay R Pitt B Opposite predictive value of pulse pressure and aortic pulse wave velocity on heart failure with reduced left ventricular ejection fraction: insights from an eplerenone post-acute myocardial infarction heart failure efficacy and survival study (Ephesus) substudy. *Hypertension.* (2014) 63:105–11. 10.1161/hypertensionaha.113.02046 24126172

[34] Franklin SS Gustin WT Wong ND Larson MG Weber MA Kannel WB hemodynamic patterns of age-related changes in blood pressure. The Framingham heart study. *Circulation.* (1997) 96:308–15. 10.1161/01.cir.96.1.308 9236450

[35] Khattar RS Swales JD. Pulse pressure and prognosis. *Heart.* (2001) 85:484–6. 10.1136/heart.85.5.484 11302985 PMC1729729

[36] Hope SA Hughes AD. Drug effects on the mechanical properties of large arteries in humans. *Clin Exp Pharmacol Physiol.* (2007) 34:688–93. 10.1111/j.1440-1681.2007.04661.x 17581231

[37] Vaitkevicius PV Lane M Spurgeon H Ingram DK Roth GS Egan JJ A cross-link breaker has sustained effects on arterial and ventricular properties in older rhesus monkeys. *Proc Natl Acad Sci USA.* (2001) 98:1171–5. 10.1073/pnas.98.3.1171 11158613 PMC14727

[38] Laurent S Boutouyrie P Lacolley P. Structural and genetic bases of arterial stiffness. *Hypertension.* (2005) 45:1050–5. 10.1161/01.HYP.0000164580.39991.3d 15851625

[39] Brugts JJ Ninomiya T Boersma E Remme WJ Bertrand M Ferrari R The consistency of the treatment effect of an ace-inhibitor based treatment regimen in patients with vascular disease or high risk of vascular disease: a combined analysis of individual data of advance, Europa, and progress trials. *Eur Heart J.* (2009) 30:1385–94. 10.1093/eurheartj/ehp103 19346520

[40] Williams B Lacy PS Thom SM Cruickshank K Stanton A Collier D Differential impact of blood pressure-lowering drugs on central aortic pressure and clinical outcomes: principal results of the conduit artery function evaluation (cafe) study. *Circulation.* (2006) 113:1213–25. 10.1161/circulationaha.105.595496 16476843

[41] Kengne AP Czernichow S Huxley R Grobbee D Woodward M Neal B Blood pressure variables and cardiovascular risk: new findings from advance. *Hypertension.* (2009) 54:399–404. 10.1161/hypertensionaha.109.133041 19470869

[42] Safar ME Blacher J Jankowski P Arterial stiffness, pulse pressure, and cardiovascular disease-is it possible to break the vicious circle? *Atherosclerosis* (2011) 218:263–71. 10.1016/j.atherosclerosis.2011.04.039 21621778

