# Supplementary material for: Association Between Pulse Pressure With All-Cause and Cardiac Mortality in Acute Coronary Syndrome: An Observational Cohort Study
Source: Front Cardiovasc Med. 2022 Jul 13;9:930755. doi: 10.3389/fcvm.2022.930755 (PMC9325995; doi:10.3389/fcvm.2022.930755)

Supplemental materials for

Association between Pulse Pressure with All-cause and Cardiac Mortality in Acute Coronary Syndrome: An Observational Cohort Study

Supplemental Table 1. Multivariable Cox regression analysis for 1-year all-cause mortality in whole cohort, UAP patients, and AMI patients

Variables	Whole Cohort		UAP		AMI	
	HR (95% CI)	*P* value	HR (95% CI)	*P* value	HR (95% CI)	*P* value
**PP (spline)**	-	**< 0.001**	**-**	**0.030**	**-**	**0.010**
**Age**	1.04 (1.02 to 1.05)	**< 0.001**	1.05 (1.02 to 1.08)	**0.002**	1.03 (1.01 to 1.05)	**0.004**
**Gender**	0.88 (0.65 to 1.18)	0.393	0.97 (0.59 to 1.60)	0.897	0.84 (0.57 to 1.22)	0.352
**BMI**	0.91 (0.88 to 0.95)	**< 0.001**	0.91 (0.85 to 0.97)	**0.004**	0.94 (0.90 to 0.99)	**0.010**
**Previous diabetes mellitus**	0.90 (0.65 to 1.25)	0.540	1.36 (0.78 to 2.34)	0.276	0.77 (0.51 to 1.18)	0.232
**Previous hypertension**	1.32 (0.94 to 1.85)	0.108	1.16 (0.61 to 2.21)	0.657	1.56 (1.04 to 2.35)	**0.031**
**Previous MI**	1.35 (0.94 to 1.94)	0.104	1.83 (0.99 to 3.38)	0.052	1.26 (0.79 to 2.00)	0.328
**Current smoking**	1.17 (0.84 to 1.63)	0.347	1.01 (0.54 to 1.89)	0.979	1.09 (0.73 to 1.61)	0.681
**Atrial fibrillation**	1.69 (1.18 to 2.40)	**0.004**	2.04 (1.12 to 3.72)	**0.021**	1.41 (0.90 to 2.19)	0.134
**Previous DAPT**	1.32 (0.94 to 1.85)	0.106	1.23 (0.69 to 2.19)	0.475	1.33 (0.87 to 2.02)	0.188
**Heart rate**	1.02 (1.01 to 1.02)	**< 0.001**	1.01 (1.00 to 1.03)	0.055	1.01 (1.00 to 1.02)	**0.015**
**LVEF < 50%**	3.02 (2.19 to 4.15)	**< 0.001**	2.11 (1.07 to 4.15)	**0.032**	2.16 (1.50 to 3.09)	**< 0.001**
**HbA1C**	1.18 (1.06 to 1.31)	**0.002**	1.14 (0.92 to 1.41)	0.245	1.14 (1.01 to 1.29)	**0.029**
**LDL-C**	1.07 (0.91 to 1.28)	0.410	1.00 (0.72 to 1.38)	0.984	1.06 (0.86 to 1.29)	0.602
**eGFR**	0.98 (0.98 to 0.99)	**< 0.001**	0.97 (0.96 to 0.98)	**<0.001**	0.99 (0.98 to 1.00)	**0.034**
**Peak value of cTnI**	1.00 (0.98 to 1.02)	0.893	1.19 (0.77 to 1.84)	0.426	0.98 (0.95 to 1.00)	0.099
**LM lesion**	1.28 (0.80 to 2.06)	0.299	2.08 (1.00 to 4.30)	**0.049**	0.80 (0.41 to 1.54)	0.498
**Multi-vessel lesion**	0.64 (0.43 to 0.97)	**0.037**	1.13 (0.60 to 2.11)	0.704	0.51 (0.29 to 0.90)	**0.020**

**Chronic total occlusion lesion**	1.40 (0.94 to 2.10)	0.099	0.73 (0.32 to 1.65)	0.451	1.50 (0.91 to 2.48)	0.115
**PCI treatment**	0.61 (0.39 to 0.95)	**0.031**	0.49 (0.22 to 1.09)	0.081	0.57 (0.32 to 1.02)	0.057
**In-hospital DAPT**	1.49 (1.05 to 2.11)	**0.026**	1.70 (0.86 to 3.35)	0.124	0.83 (0.56 to 1.25)	0.376
**In-hospital ACEI/ARB**	0.67 (0.50 to 0.89)	**0.006**	1.09 (0.66 to 1.80)	0.726	0.47 (0.32 to 0.68)	**< 0.001**
**In-hospital Statins**	0.47 (0.35 to 0.64)	**< 0.001**	0.78 (0.42 to 1.45)	0.440	0.48 (0.33 to 0.71)	**< 0.001**

Abbreviation: ACEI, angiotensin-converting enzyme inhibitor; ARB, angiotensin receptor blocker; BMI, body mass index; cTnI, cardiac troponin I; DAPT, dual-antiplatelet therapy; eGFR, estimated glomerular filtration rate; HbA1C, glycosylated hemoglobin; LDL-C, low-density lipoprotein cholesterol; LM, left main coronary artery; LVEF, left ventricular ejection fraction; MI, myocardial infarction; PCI, percutaneous coronary intervention; PP, pulse pressure.

Supplemental Table 2. Multivariable Cox regression analysis for 1-year cardiac mortality in whole cohort, UAP patients, and AMI patients

Variables	Whole Cohort		UAP		AMI	
	HR (95% CI)	*P* value	HR (95% CI)	*P* value	HR (95% CI)	*P* value
**PP (spline)**	-	**< 0.001**	**-**	**0.046**	**-**	**0.017**
**Age**	1.04 (1.02 to 1.06)	**< 0.001**	1.04 (1.00 to 1.08)	**0.075**	1.04 (1.02 to 1.06)	**< 0.001**
**Gender**	0.97 (0.68 to 1.39)	0.879	1.60 (0.79 to 3.21)	0.191	0.79 (0.52 to 1.22)	0.292
**BMI**	0.90 (0.86 to 0.94)	**< 0.001**	0.85 (0.78 to 0.94)	**< 0.001**	0.94 (0.89 to 0.99)	**0.025**
**Previous diabetes mellitus**	1.05 (0.72 to 1.55)	0.790	2.09 (0.98 to 4.46)	0.058	0.89 (0.55 to 1.43)	0.620
**Previous hypertension**	1.21 (0.81 to 1.80)	0.351	0.98 (0.41 to 2.34)	0.966	1.38 (0.88 to 2.18)	0.159
**Previous MI**	1.48 (0.97 to 2.26)	0.070	2.76 (1.25 to 6.13)	**0.012**	1.36 (0.82 to 2.28)	0.237
**Current smoking**	1.03 (0.68 to 1.56)	0.885	0.51 (0.17 to 1.51)	0.224	1.03 (0.65 to 1.62)	0.916
**Atrial fibrillation**	1.74 (1.15 to 2.62)	**0.009**	3.53 (1.64 to 7.56)	**0.001**	1.26 (0.77 to 2.08)	0.361
**Previous DAPT**	1.10 (0.72 to 1.69)	0.651	0.77 (0.33 to 1.81)	0.546	1.18 (0.72 to 1.93)	0.520
**Heart rate**	1.01 (1.01 to 1.02)	**0.001**	1.01 (0.99 to 1.03)	0.310	1.01 (1.00 to 1.02)	**0.026**
**LVEF < 50%**	3.88 (2.66 to 5.66)	**< 0.001**	2.94 (1.19 to 7.25)	**0.019**	2.40 (1.59 to 3.62)	**< 0.001**
**HbA1C**	1.16 (1.02 to 1.31)	**0.020**	1.16 (0.88 to 1.54)	0.299	1.11 (0.96 to 1.27)	0.151
**LDL-C**	0.96 (0.77 to 1.19)	0.711	0.83 (0.52 to 1.32)	0.422	0.97 (0.77 to 1.24)	0.831
**eGFR**	0.99 (0.98 to 0.99)	**< 0.001**	0.98 (0.96 to 1.00)	**0.032**	0.99 (0.98 to 1.00)	0.076
**Peak value of cTnI**	1.01 (0.99 to 1.04)	0.276	1.23 (0.78 to 1.94)	0.365	0.99 (0.96 to 1.02)	0.508
**LM lesion**	1.48 (0.84 to 2.59)	0.175	3.11 (1.20 to 8.03)	**0.019**	0.88 (0.41 to 1.88)	0.737
**Multi-vessel lesion**	0.47 (0.27 to 0.80)	**0.006**	1.20 (0.49 to 2.95)	0.696	0.36 (0.18 to 0.72)	**0.004**
**Chronic total occlusion lesion**	1.89 (1.14 to 3.12)	**0.013**	1.00 (0.35 to 2.80)	0.995	1.85 (1.01 to 3.40)	**0.046**
**PCI treatment**	0.82 (0.46 to 1.44)	0.488	0.37 (0.13 to 1.08)	0.070	0.84 (0.42 to 1.68)	0.627
**In-hospital DAPT**	1.28 (0.83 to 1.97)	0.258	2.43 (0.97 to 6.06)	0.057	0.64 (0.40 to 1.02)	0.060
**In-hospital ACEI/ARB**	0.65 (0.46 to 0.91)	**0.014**	1.12 (0.56 to 2.23)	0.748	0.49 (0.32 to 0.75)	**0.001**
**In-hospital Statins**	0.37 (0.26 to 0.52)	**< 0.001**	0.43 (0.21 to 0.89)	**0.023**	0.46 (0.30 to 0.71)	**< 0.001**

Abbreviation: ACEI, angiotensin-converting enzyme inhibitor; ARB, angiotensin receptor blocker; BMI, body mass index; cTnI, cardiac troponin I; DAPT, dual-antiplatelet therapy; eGFR, estimated glomerular filtration rate; HbA1C, glycosylated hemoglobin; LDL-C, low-density lipoprotein cholesterol; LM, left main coronary artery; LVEF, left ventricular ejection fraction; MI, myocardial infarction; PCI, percutaneous coronary intervention; PP, pulse pressure.

Supplemental Table 3. 1-year mortality according to PP categories in patients with PP < 50 mmHg

PP categories	n (%)	All-cause death, n (%)	Cardiac death, n (%)
**SBP ≥ 120 mmHg & DBP ≥ 70 mmHg**	1,618 (45.87)	26 (1.61)	14 (0.87)
**SBP < 120 mmHg & DBP ≥ 70 mmHg**	1,096 (31.07)	28 (2.55)	22 (2.01)
**SBP < 120 mmHg & DBP < 70 mmHg**	813 (23.06)	27 (3.32)	19 (2.34)

Abbreviation: DBP, diastolic blood pressure; SBP, systolic blood pressure; PP, pulse pressure.

Supplemental Figure 1. Kaplan-Meier curves according to PP categories in patients with PP < 50 mmHg

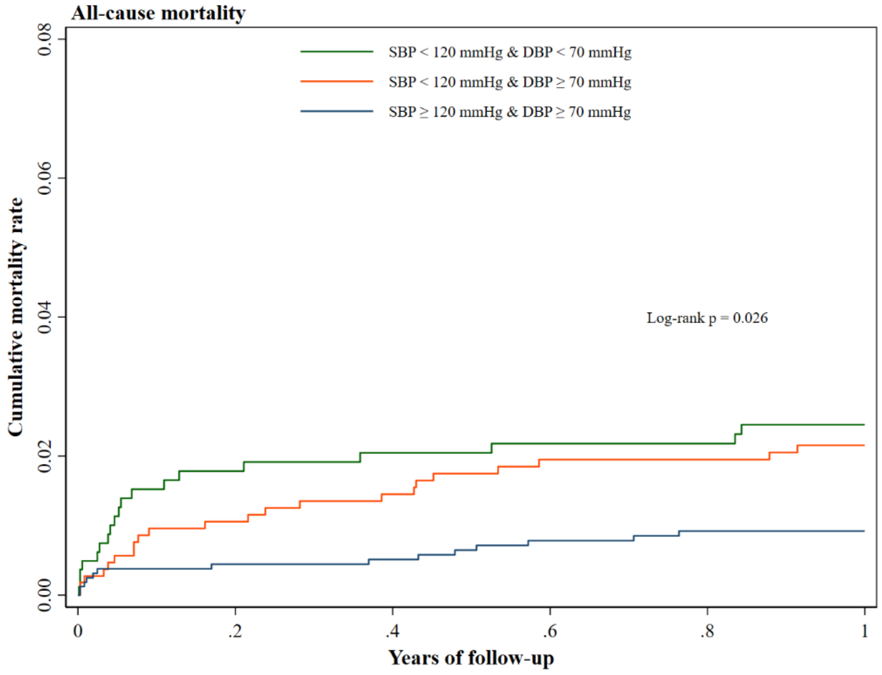

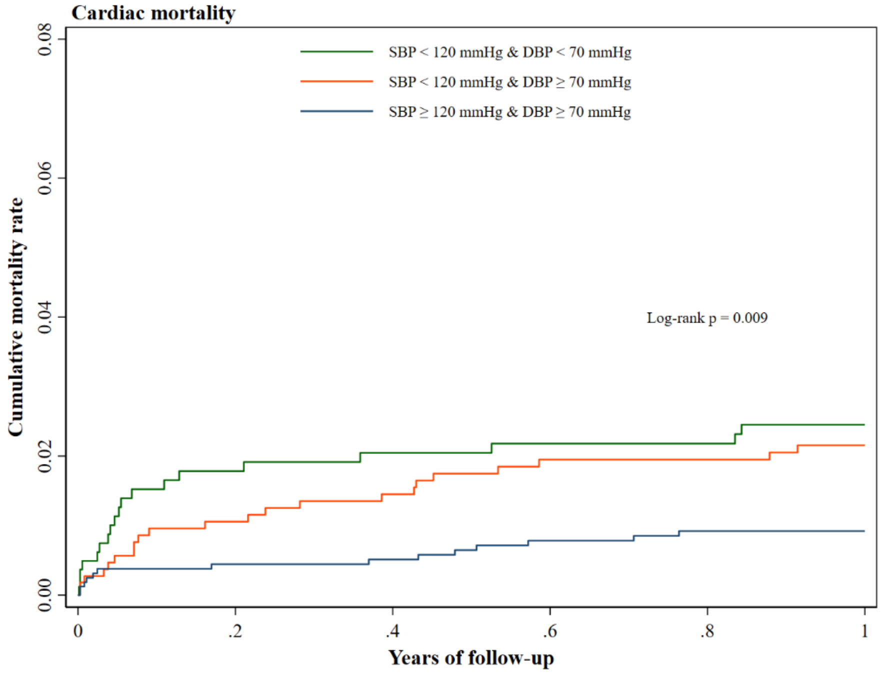

Supplement: Supplementary file 1 [file Data_Sheet_1.pdf]
